# Protein tyrosine phosphatase, receptor type B is a potential biomarker and facilitates cervical cancer metastasis via epithelial-mesenchymal transition

**DOI:** 10.1080/21655979.2021.1968250

**Published:** 2021-09-13

**Authors:** Zhuo-Ya Huang, Peng-Juan Liao, Ying-xia Liu, Ming Zhong, Ai-hua Sun, Xiao-cong Jiang, Xiu-Ping Wang, Min Zhang

**Affiliations:** a Department of Pathology, The Huizhou Municipal Central Hospital, Huizhou, Guangdong, China; b Department of Pathology, The First Hospital of Huizhou, Huizhou, Guangdong, China; c Department of Oncology, The Huizhou Municipal Central Hospital, Huizhou, Guangdong, China; d Department of Radiotherapy, The Huizhou Municipal Central Hospital, Huizhou, Guangdong, China; e Department of Outpatient, The Huizhou Municipal Central Hospital, Huizhou, Guangdong, China

**Keywords:** PTPRB, TCGA database, cervical cancer, biomarker, metastasis

## Abstract

Cervical cancer (CC) is one of the most common malignant tumors. This study analyzed the impact of protein tyrosine phosphatase, receptor type B (PTPRB) on malignant behavior of CC and explored its possible molecular mechanism. RT-PCR, western blot and Immunohistochemistry were applied to examine the expression of PTPRB in CC specimens and cells. Aberrant PTPRB expression in CC and survival outcomes were constructed using The Cancer Genome Atlas (TCGA) database and tissue microarray cervical squamous cell carcinoma cohort. Cultured human CC cells were assayed for viability, apoptosis, migration, and invasion in vitro and in vivo. Kyoto Encyclopedia of Genes and Genomes (KEGG) assays and gene set enrichment analysis (GSEA) assays were used to delve into PTPRB-related pathways using TCGA datasets. The levels of proteins associated with the epithelial–mesenchymal transition (EMT) pathway and modulated by PTPRB were examined through Western blot. We found that the levels of PTPRB in CC tissues and cells were distinctly up-regulated. PTPRB was also an unfavorable prognostic factor for CC patients. Functionally, PTPRB knockdown exhibits tumor-suppressive function via reducing cell proliferation and metastasis and inducing cell apoptosis. KEGG assays and GSEA assays suggested PTPRB overexpression was associated with several tumor-related pathways. The results of Western blot assays suggested that N-cadherin was decreased in the PTPRB-knockdown CC cells, while E-cadherin was increased. Overall, PTPRB is highly expressed in CC and can effectively enhance the proliferation, metastasis and EMT process of tumor cells. PTPRB is expected to be a therapeutic target for CC.

## Introduction

As the fourth leading cause of tumor-related deaths in females globally, cervical cancer (CC) is the fourth most common diagnosed cancer [1]. According to latest documents, >530,000 new cases are reported per year [2]. Even though the advancements of diagnostic programs and effective operations in the last decade have improved the long-term survivals of CC patients, for advanced-stage CC patients, their poor prognosis was frequently observed and survival rates for many CC patients remain exceedingly low [3–5]. Thus, it is of great importance to achieve an advanced understanding of the biological mechanisms underlying CC and search novel diagnostic/prognostic biomarkers for this disease.

Protein tyrosine phosphatase, receptor type B (PTPRB), located on chromosome 12q15, is also known as RPTPβ and VE-PTP [6]. Belonging to the protein tyrosine phosphatase family, PTPRB consists of a single intracellular catalytic domain with C-terminal phosphorylation sites, a transmembrane domain and an extracellular domain with multiple fibronectin type III-like domains [7,8]. PTPRB has been functionally demonstrated to be involved in the formation and remodeling of vascellum [9,10]. In recent years, more and more studies have reported the distinct dysregulation of PTPRB in many types of tumors according to Chip sequencing [11,12]. In addition, in several tumors, functional assays have been applied to demonstrate the tumor-related effects of PTPRB on tumor progression. For instance, overexpression of PTPRB suppressed the growth and migration of bronchogenic carcinoma cells via increasing Src phosphorylation, and predicted a poor prognosis of patients with lung cancer [13]. However, in colorectal carcinoma, PTPRB displayed an anti-oncogene effect, suggesting its multiple functions in different types of tumors [14–16]. To date, whether PTPRB expression was dysregulated in CC and its potential function had not been investigated.

In this study, we aimed to explore the expressing pattern and function of PTPRB in CC. We first confirmed that PTPRB was overexpressed in CC patients. Then, we explored the prognostic value of PTPRB upregulation in CC patients and its potential function in tumor behavior. Collectively, our data suggested the biological functions of PTPRB in CC for the first time and provided evidences that PTPRB may represent a biomarker for CC screening and be applied as an RNAi-based target for CC.

## Materials and methods

### Human tissue specimen collection

A total of 30 pairs of tumors and adjacent normal cervical tissues were surgically resected from CC patients admitted to our hospital from January 2018 to January 2020. All of the patients did not receive preoperative radiotherapy, chemotherapy, targeted therapy and immunotherapy. Based on histopathological data, two clinicians independently reported the diagnosis of all patients. All tissue samples were stored at −80°C immediately after the resection. Informed consent was obtained from the subjects, and the protocol was approved by the Ethics Committee of The Huizhou Municipal Central Hospital. All experiments were conducted under the rule of the Declaration of Helsinki.

### Tissue microarrays

The Gene Expression Omnibus DataSets is a publicly available database, which provided microarray data for users’ researches. GSE63514 (including 28 CC samples and 24 non-tumor samples) were applied to study the expressions of PTPRB in tumor and non-tumor specimens [17]. The sequencing data and corresponding clinical data of CC patients were provided using The Cancer Genome Atlas (TCGA) datasets (https://tcga-data.nci.nih.gov/tcga/).TMA) cohort (85 tumor samples) including survival data were purchased from Outdo Biotech (Shanghai, China) (Outdo cohort).

### Cell culture and transfection

Human CC cell lines (C33A, CASKI, Siha, Hela and HT-3) and skin epithelial cells Hacat cells were obtained from the Cell Bank of the Chinese Academy of Sciences. All cells were cultured in DMEM (L340, Aiyan Biology, Pudong, Shanghai, China) with 10% Fetal Bovine Serum (FBS, GIBCO, BSK, Guandong, Guandong, China) at a 37°C, 5% CO_2_ incubator. A short hairpin RNA (shRNA) lentiviral vector (sh-PTPRB#1 and sh-PTPRB#2) was structured to silence PTPRB expressions in CC cells. Based on the products guide, the transfection was carried out by the use of Lipofectamine 3000 transfection reagent (L3000015, Invitrogen, Ruinuode, Technology, Suzhou, Jiangsu, China). Western blotting and RT-PCR were applied to demonstrate the rates of inhibition of PTPRB expressions.

### Quantitative real-time PCR (qRT-PCR)

By the use of TRIzol reagent (B0201, HaiGene, Haerbing, Heilongjiang, China), our group collected total RNA from human specimens and cells following products guide. Reverse transcription of RNA was applied to obtain cDNA templates by applying Super M-MLV reverse transcriptase. According to the direction of the reagents, ABI Prism 7900HT (Applied Biosystems) was used to perform RT-PCR for the examination of the related factors under the following thermocycler conditions: 95°C for 2 min, and 40 cycles of 95°C for 5 s and 60°C for 30 s. Expression levels of PTPRB were normalized using the 2^−ΔΔCt^ methods relative to GAPDH. The primers to amplify PTPRB were 5’-GGGCTCACCCTGTAACTTTAGC-3’ (forward) and 5’-TCTATCCGAAAGGTAGGGCAC-3’ (reverse), and for GAPDH were 5’-GGAGCGAGATCCCTCCAAAAT-3’ (forward) and 5’-GGCTGTTGTCATACTTCTCATGG-3’ (reverse).

### Western blot assays

Western blot assays were carried out as previously described [18]. Antibodies (1:1000) for PTPRB, N-cadherin, E-cadherin and GAPDH were purchased from Sigma-Aldrich (Pudong, Shanghai, China).

### Immunohistochemistry (IHC)

The neoplasms were cut into 4-μm-thick sections. Subsequently, the immunohistochemistry (IHC) assays were carried out, as described previously [19]. Briefly, after being fixed, dehydrated and embedded, the sections were immersed in sodium citrate (T0117, Baomanbio, Xuhui, Shanghai, China), followed by heat for antigen retrieval. Subsequently, anti-PTPRB, anti-E-cadherin, anti-N-cadherin, anti-KI67 and secondary antibody were used to incubate the collected specimens. A microscope was applied to examine the staining results.

### Immunofluorescence

Cells (C33A and CASKI) were fixed in 4% paraformaldehyde for 15 min at 25°C. Then, 2% BSA (PC0001-1 ml, Acmec, Jizhibio, Fengxian, Shanghai, China) was applied to block the cells 1 h at 25°C. Primary antibody PTPRB (1:200) was incubated at 4°C overnight and FITC-labeled secondary anti-mouse antibody was incubated for 1 h at 25°C. DAPI was applied to counterstain Nuclei. Finally, the cells were sealed, and images were acquired using a confocal microscope (magnification, ×100).

### Cell counting kit-8 (CCK-8) assay

The oncogenic roles of PTPRB knockdown on the viabilities of C33A and CASKI cells were determined using CCK-8 (CP002, SAB, Nanjing, China). In short, after RPMI-1640 medium supplemented with DNR, C33A and CASKI cells (1 × 10^4^) were cultured in the above medium. Subsequently, to each well, the CCK-8 solution (10 μl) was added, followed by incubation for 2 h. Finally, the absorbance was examined in each individual well at 450 nm, followed by the statistics of the growth curve with the collected data.

### Colony formation assay

C33A and CASKI cells were plated in 12-well plates and incubated in DMEM with 10% FBS with 5% CO_2_ at 37°C. Six days later, the cells were fixed, followed by stain by the use of 0.1% crystal violet. Ultimately, the number of colonies (≥50 cells) was calculated manually. More than three independent repeats were performed in this assay.

### EdU assay

Using Cell-Light^#^™ EdU-Apollo569 In-Vitro# Imaging Kit (RiboBio, Haidian, Beijing, China), EdU assays were carried out for the determination of proliferating abilities of 1 × 10^4^ cells after PTPRB knockdown. After EdU labeling, a fluorescence microscope (Olympus, Dalian, Niaoning, China) was applied to visualize EdU-positive cells.

### TUNEL staining

In Situ Cell Apoptosis Detection Kit (Roche, Hangzhou, Zhejiang, China) was applied to examine apoptotic cells. Then, phosphate buffered saline (PBS) was used to rinse the collected cells on coverslips. After TUNEL staining reagent and DAPI dye were used for double-staining, a fluorescent microscope was applied to obtain fluorescence pictures. The experiment was repeated three times.

### Transwell assay

For migration assays, Transwell chambers (Yanhui Biology, Jiading, Shanghai, China) were placed in 24-well plates. C33A and CASKI cells (1 × 10^5^) were placed in the uncoated upper chamber without serum, and the lower chamber was full of 10% FBS to induce cellular movements of the membrane. After incubation for 24 h at 37°C, the membrane was fixed with 4% paraformaldehyde for 25 min at room temperature. 0.1% crystal violet solution was used to stain the cells that have moved to the lower surface of the membrane for 30 min. Subsequently, the cells were counted under a microscope. For cellular invasion, the filter was first covered by the use of diluted Matrigel (BD, Haidian, Beijing, China), and the other procedures were in line with the above progresses.

### Wound healing assay

Cells (2 × 10^5^) were seeded into 12-well plates and cultured. A 200-μl pipette tip helped to generate wounds in a cell monolayer at 80% confluence. Then, the cells were washed and recultured with a serum-free medium. The effects of PTPRB silence on the migrative abilities of CC cells was determined by adding a medium containing 20 μg/ml mitomycin C. At 0 and 48 h after wounding, healing was assessed after pictures were taken.

### Animal experiments

Female BALB/c nude mice (Kawensi Biology, Changzhou, Jiangsu, China) were applied for CC model. PTPRB-knockdown or control C33A cells were injected into subcutaneous flanks of 5-week-old nude mice (n = 5 per group). All mice were sacrificed 5 weeks after injection and xenografts were taken out for weighting. Tumor volumes were calculated using the following standard formula: length × width^2^/2. The incidence of metastatic nodes in lungs was determined by the presence of macroscopic lesions on the surface of the lung. The animal-related protocol was approved by the Animal Research Ethics Committee of The Huizhou Municipal Central Hospital. Our group did our best to minimize animal suffering

### Gene set enrichment analysis

Based on the median expression of PTPRB in all samples, 279 cervical squamous cell carcinoma(CESC) samples in TCGA were divided into two groups (high and low). To identify the significantly altered Kyoto Encyclopedia of Genes and Genomes (KEGG) pathways, we performed gene set enrichment analysis (GSEA) between the high-expressing and low-expressing groups using the Java GSEA software. A nominal (NOM) p-value <0.05 was chosen as the cutoff criterion.

### Statistical analysis

SPSS (version 19.0, SPSS Co., USA) and R software (R version 3.7.1) were used to complete the statistical assays. The results are expressed as the median (range) or the mean ± SD. For statistical comparisons among different groups, two-tailed Student’s t-test or one-way ANOVA was applied. Survival analyses were performed in R environment using survival and survminer packages. A p-value of <0.05 was considered statistically significant.

### Results

#### PTPRB was overexpressed in CC

**Figure 1.**
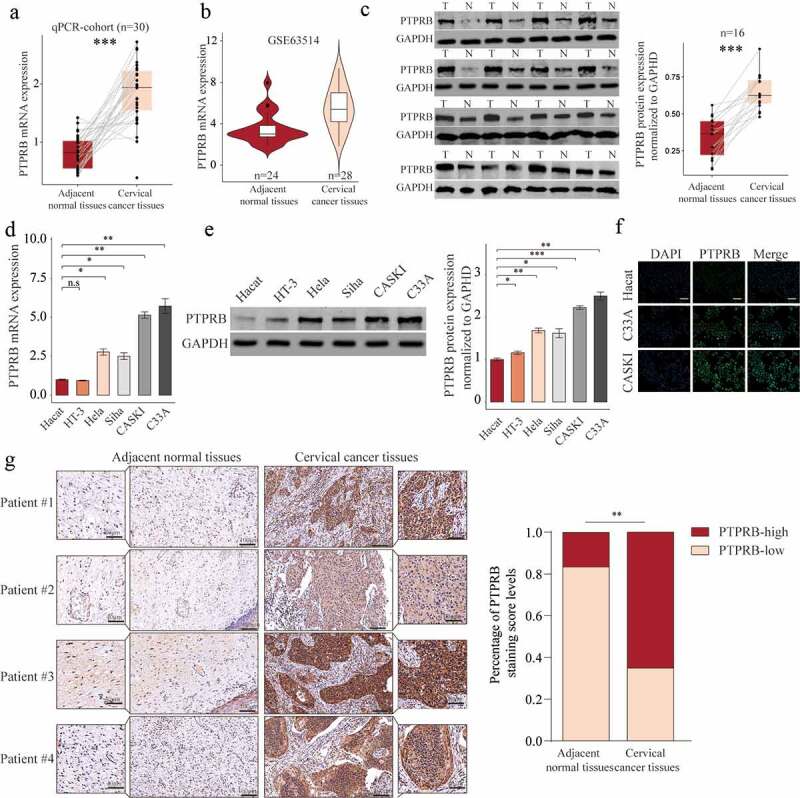
PTPRB expression was distinctly increased in CC. (a, b) PTPRB expressions in CC specimens compared with normal specimens in our cohort(a) and GSE63514 datasets(b). (c) Western blot assays were applied to evaluate the protein levels of PTPRB in 8 pairs of CC specimens and the adjacent non-tumor specimens. (d,e)The expression of PTPRB at mRNA and protein levels in five CC cells and Hacat cells. (f) Immunofluorescence determined the expressions of PTPRB in C33A and CASKI cells. (g) The levels of PTPRB in cancer and non-tumor samples from our patients were analyzed by IHC staining. The experiment was performed three times with three replications. *p < 0.05, **p < 0.01, ***p < 0.001

**Figure 2.**
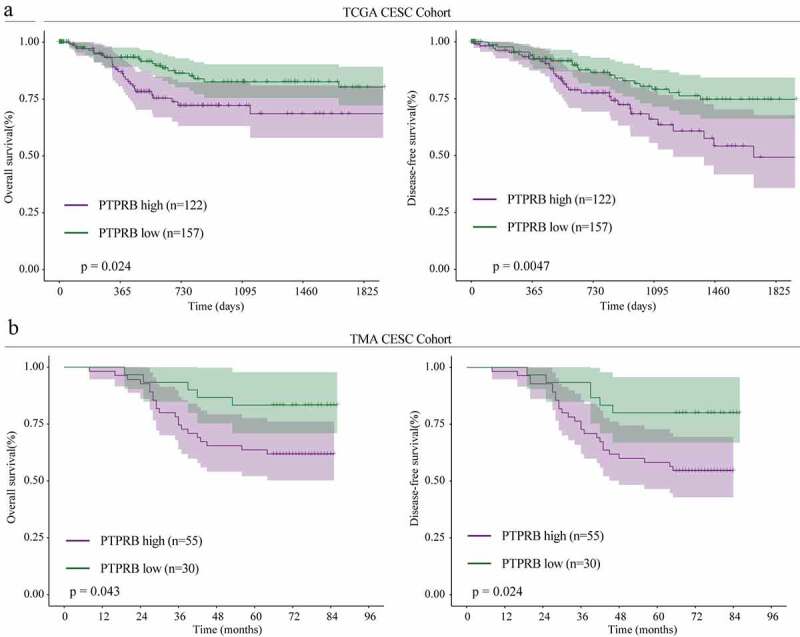
The prognostic value of PTPRB dysregulation in CC patients. The relationships between PTPRB expressions and survival time of CC patients from TCGA CESC cohort(a) and TMA CESC cohort(b) analyzed using the Kaplan–Meier method

In order to explore whether PTPRB was dysregulated in CC, we performed RT-PCR in 30 samples and found that PTPRB expression was distinctly upregulated in CC specimens compared with the matched non-tumor specimens (Figure 1a), which was further confirmed by amazing GSE63514 (Figure 1b). Western blot assays also confirmed the protein expressions of PTPRB in CC specimens were distinctly higher than those in adjacent normal specimens (Figure 1c). Furthermore, PTPRB expression levels are markedly increased in CC cells (HT-3, Hela, Siha, CASKI and C33A) compared with Haca cells (Figure 1d and 1e), which was also confirmed using Immunofluorescence (Figure 1f). Given the relative higher expression of PTPRB in CASKI and C33A cell lines, we chose them for further functional experiments. Besides, we performed IHC, finding that PTPRB protein displayed distinctly higher expression in CC specimens than in non-tumor specimens(Figure 1g). These findings that PTPRB may be a positive factor for CC. Thus, we further analyzed the clinical significance of PTPRB expression in CC patients. As shown in Figure 2a, patients with high PTPRB expressions exhibited a shorter OS (p = 0.024) and DFS (p = 0.0047) than those with low PTPRB expressions based on TCGA datasets, which was also further confirmed using TMA CESC Cohort (n = 85) (Figure 2b). Overall, our findings suggested PTPRB as a positive factor involved in the outcomes of CC patients.

### PTPRB knockdown suppressed the proliferation, migration and invasion of CC cells

**Figure 3.**
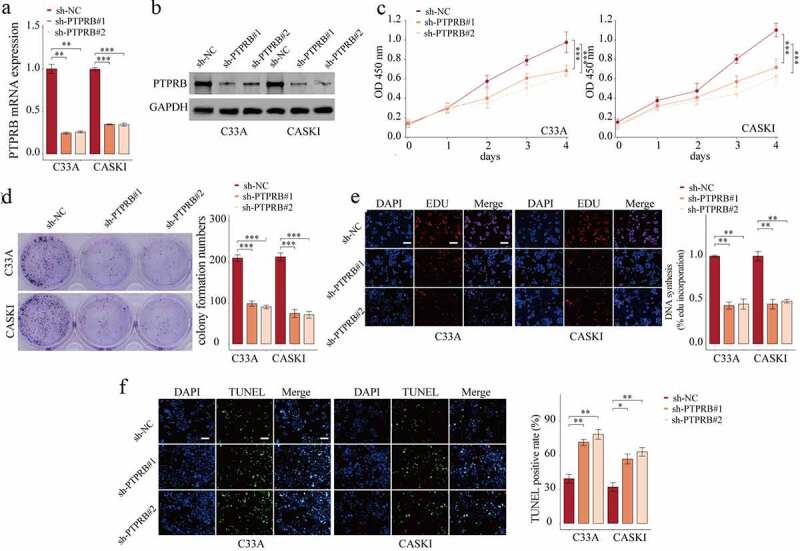
Silencing of PTPRB suppressed the proliferation of CC cells in vitro. (a,b) Silence of PTPRB was verified by qRT-PCR and western blot. (c) Cell proliferation rate of CC cells transfected with sh-PTPRB#1 or sh-PTPRB#2 by CCK-8. (d, e) Proliferation of CC cells with PTPRB silence from Colony formation assay and EdU assay. (f) TUNEL staining was performed to observe cell apoptosis of C33A and CASKI transfected with sh-NC, sh-PTPRB#1 or sh-PTPRB#2. The experiment was performed three times with three replications. *p < 0.05, **p < 0.01, ***p < 0.001

**Figure 4.**
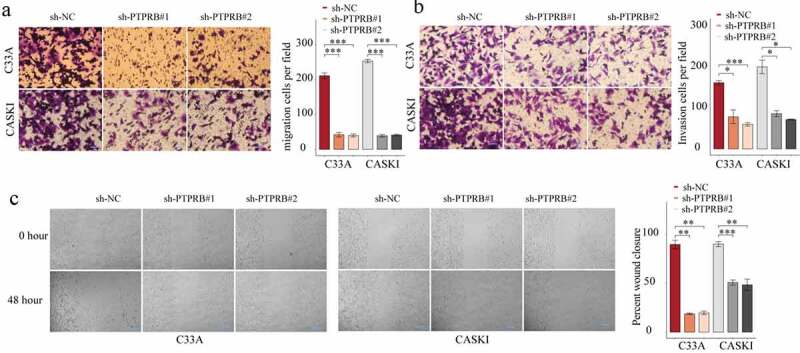
Silencing of PTPRB suppressed CC metastasis. (a, b) Transwell assays evaluated cellular migration and invasion. (c) Cell migration was examined by wound healing assays. The experiment was performed three times with three replications. *p < 0.05, **p < 0.01, ***p < 0.001

To elucidate the effects of PTPRB on C33A and CASKI cells, we studied the effects of PTPRB silence on proliferative and metastatic abilities. First, we tested the efficiency of sh-PTPRB#1 and sh-PTPRB#2. The results show that PTPRB was effectively decreased in C33A and CASKI cells (Figure 3a and 3b). Applying the CCK-8 assays, proliferation of CC cells was shown to be distinctly decreased with the silence of PTPRB (Figure 3c); these results were similar to those obtained from the colony formation experiment and EdU assay (Figure 3d and 3e). Moreover, PTPRB downregulation distinctly promoted apoptosis in C33A and CASKI cells (Figure 3f). Also, PTPRB silence distinctly reduced cell migration and invasion (Figure 4a- 4c).

### PTPRB knockdown delayed the development and metastasis of CC in vivo

**Figure 5.**
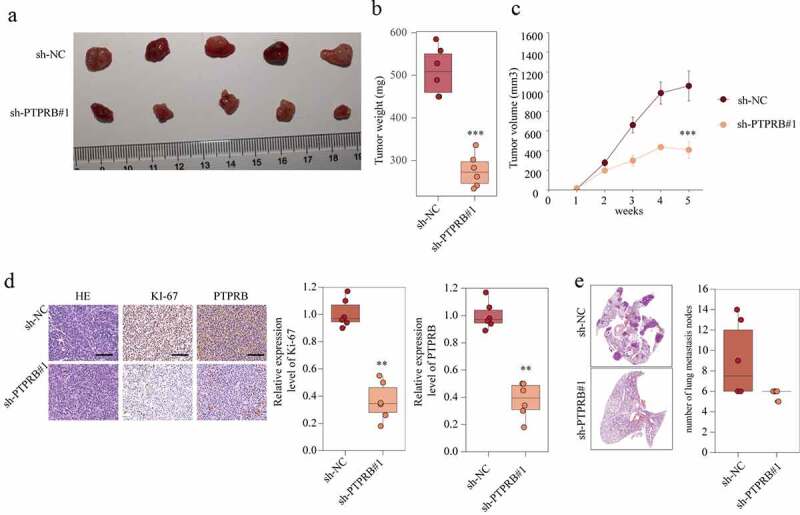
PTPRB knockdown delayed the development and metastasis of CC in vivo. (a) Representative images of xenograft tumors of each group. (b) The weight of tumors obtained from two groups was measured and shown. (c) Growth curves of xenograft tumors of each group. (d) Expression levels of KI67 in two groups were analyzed by IHC staining. (e) Histopathological changes of lung tissues and lymph node metastasis in xenograft mice transfected with sh-PTPRB#1 or sh-NC. The experiment was performed three times with three replications. **p < 0.01, ***p < 0.001

To further evaluate the effect of PTPRB on tumor growth, an in vivo C33A cell line-derived xenograft tumor model was conducted in nude mice. The tumor weight was distinctly decreased in the sh-PTPRB group compared to the NC group (Figure 5a and Figure 5b). These results were correlated with the tumor volume in the fifth week (Figure 5c). Moreover, PTPRB and Ki-67 in the tumors of the sh-PTPRB group were distinctly weaker than those in the tumors of the sh-NC group (Figure 5d). Lymph nodes surrounding tumor xenografts were resected for HE staining. We observed that the number of lung metastasis nodes was distinctly increased in the sh-NC group compared with sh-PTPRB#1 group (Figure 5e). These data suggested that PTPRB knockdown delays the development and metastasis of CC in vivo.

### The activity of epithelial–mesenchymal transition (EMT) signal was suppressed after PTPRB knockdown

**Figure 6.**
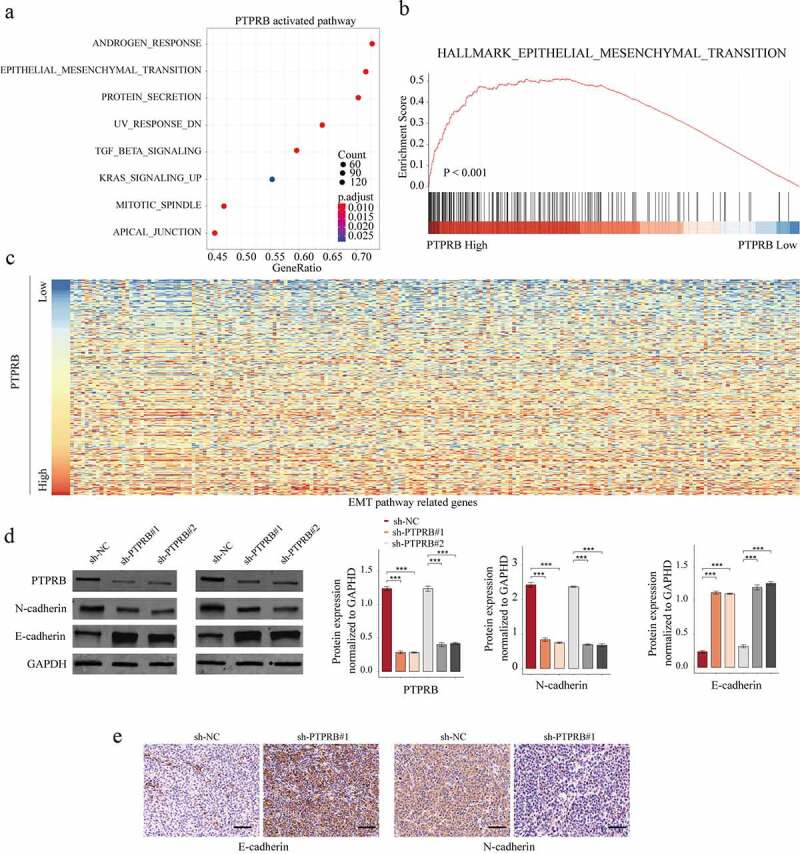
The effects of PTPRB knockdown on EMT activity. (a) KEGG analysis for the exploration of PTPRB-related pathways. (b) GSEA analysis for the enrichments of the EMT-related gene sets in CC specimens based on PTPRB levels. (c) Heat Map of EMT pathway-related genes based on PTPRB levels. (d) Western blot determined the expressions of EMT-related molecules in CASKI and C33A cells transfected with sh-PTPRB#1 or sh-NC. (e) IHC staining for the change of E-cadherin and N-cadherin levels after PTPRB silence. The experiment was performed three times with three replications. ***p < 0.001

To explore the potential mechanisms involved in the oncogenic roles of PTPRB in CC progression, our group performed KEGG assays and GSEA assays to delve into PTPRB-related pathways using TCGA datasets. We observed that the EMT signaling pathway was distinctly associated with PTPRB upregulation (Figure 6a-Figure 6c). To test this hypothesis, we perform western blot, finding that EMT pathway-related upregulation) were distinctly influenced in C33A cells transfected with sh-PTPRB#1 and sh-PTPRB#2 (Figure 6d). Further IHC assays revealed that the transfection of sh-PTPRB#1 resulted in a distinct suppression of E-cadherin and N-cadherin expression in C33A cells (Figure 6e). Overall, our findings suggested that PTPRB promoted CC metastasis via modulating the EMT pathway.

## Discussion

CC remained a major clinical challenge due to continual recurrences and cancer metastasis [20,21]. Thus, the identification of precise cancer-specific markers for early CC diagnosis was essential for the improvement of clinical outcomes of CC patients.

With the development of Chip sequencing technology in recent years, extensive amounts of sequencing data were stored in public datasets, which brought convenience for researchers exploring the novel biomarkers for various tumors [22,23]. In recent years, by the use of bioinformatic analysis, more and more possible molecules involved in CC progression have been preliminarily screened [24,25]. In this study, we first identify PTPRB as an overexpressed gene in CC by analyzing GSE63514, followed by further confirmation in our cohort using RT-PCR, western blot and immunohistochemistry. The tumor-related functions of PTPRB on cellular processes of several tumors have been previously reported. For instance, PTPRB was shown to reduce the potential ability of the growth and invasion of bronchogenic carcinoma cells through modulating the phosphorylation of the proto-oncogene tyrosine-protein kinase Src [13]. In colorectal carcinoma, PTPRB was shown to be highly expressed and promote metastasis of tumor cells via inducing EMT [14]. However, its tumor-related effects and prognostic values of PTPRB in CC have not been investigated.

In this study, we found that PTPRB expression at both mRNA and protein levels was distinctly upregulated in both CC patients and cells. Clinical assays using TGCA and TMA datasets revealed that patients with high PTPRB expressions exhibited a shorter OS and DFS, suggesting PTPRB may be involved in CC progression. Then, we performed loss-of-function assays to further explore its detailed roles, finding that knockdown of PTPRB distinctly suppressed the proliferation, migration and invasion of CC cells and promoted apoptosis. However, we only performed the growth model in vivo. In the future, the metastatic model in vivo was needed to further demonstrate the oncogenic roles of PTPRB in CC. Our data revealed PTPRB as a tumor promoter, which was not in line with its effects on lung cancer and osteosarcoma. The mechanisms involved in multiple effects of PTPRB in different types of tumors need to be further studied. Overall, these data suggested PTPRB may be used as a therapeutic target for CC.

As one of the important markers of tumorigenesis, EMT progress includes the re-differentiation of epithelial cells into mesenchymal cells, making the cell phenotype malignant [26,27]. Growing studies have demonstrated that EMT exhibited a primary power for tumor progression from cancer progression from onset to metastasis and was regulated by complex molecular mechanisms [28,29]. Its activation served as a positive factor in inducing tumor metastasis and drug resistance [30]. KEGG and GSEA assays revealed that high PTPRB was distinctly associated with EMT-related genes in TCGA datasets. Thus, we performed western blot assays to explore the influence of PTPRB knockdown on EMT-related genes, finding that knockdown of PTPRB distinctly suppressed the activity of EMT signal. However, the specific mechanism involved in the PTPRB-induced EMT in CC remains largely unclear and requires further exploration.

## Conclusion

In conclusion, we demonstrated that PTPRB acts as an oncogenic protein and promotes the proliferation, migration, invasion, and EMT of CC cells. Further studies are needed on the specific mechanism of PTPRB, showing that it mediates the EMT pathway. Thus, PTPRB may be a novel prognostic biomarker and a potential target for further preclinical studies.
